# Biomechanical effect of intertrochanteric curved varus osteotomy on stress reduction in femoral head osteonecrosis: a finite element analysis

**DOI:** 10.1186/s13018-021-02614-z

**Published:** 2021-07-23

**Authors:** Yuzhu Wang, Go Yamako, Takato Okada, Hideki Arakawa, Yoshihiro Nakamura, Etsuo Chosa

**Affiliations:** 1 Department of Orthopaedic Surgery, Faculty of Medicine, University of Miyazaki, Miyazaki, Miyazaki Japan; 2 Department of Mechanical Engineering, Faculty of Engineering, University of Miyazaki, Miyazaki, Miyazaki Japan; 3 Graduate school of Engineering, University of Miyazaki, Miyazaki, Miyazaki Japan

## Abstract

**Background:**

Intertrochanteric curved varus osteotomy (CVO) has been widely used to remove the necrotic bone away from the weight-bearing portion in the treatment of osteonecrosis of the femoral head (ONFH). However, whether all types of necrosis will benefit from CVO, in terms of the stress level, the effect of different center-edge (CE) angles of acetabulum on stress distribution of necrosis after CVO, and the relationship between the intact ratio and the stress of necrosis, has never been addressed. The purpose of the study was to evaluate the influence of CVO on the stress reduction in necrotic bone using a finite element analysis (FEA) with different CE angles.

**Methods:**

CVO finite element models of the hip joint were simulated with a lesion of 60°. The osteotomy angles were divided into four configurations (15°, 20°, 25°, and 30°), and three types (A, B, and C1) of lesions were established based on the Japanese Investigation Committee (JIC) classification. In addition, two CE angles (18° and 33°) of acetabulum were considered. The maximum and mean von Mises stress were analyzed in terms of the necrotic bone by a physiological loading condition. Moreover, the correlation of the intact ratio measured in 3D and the stress distribution after CVO was analyzed.

**Results:**

Stress reduction was obtained after CVO. For type B, the CVO angle was 20° (0.61 MPa), and for type C1, the CVO angle was 30° (0.77 MPa), if the mean stress level was close to type A (0.61 MPa), as a standard. The maximum and mean von Mises stress were higher in the CE angle of 18°models, respectively. The intact ratio measured in 3D had a good negative correlation with stress after CVO and had more influence on stress distribution in comparison to other geometric parameters.

**Conclusions:**

For making decisions about the biomechanics of CVO, a CVO angle of > 20° was recommended for type B and > 30° was safe for type C1. The risk of progressive collapse was increased in the insufficient situation of the weight-bearing portion after CVO. The intact ratio could provide information about clinical outcomes and stress distribution after CVO.

## Background

Osteonecrosis of the femoral head (ONFH) is a progressive pathological process that occurs due to multiple factors affecting the blood supply of femoral head [1]. Among the preservation procedures for the treatment of ONFH, intertrochanteric curved varus osteotomy (CVO) is a proximal femoral osteotomy technique that is widely used in Japan [2–12]. It is performed with the aim of removing necrotic regions of the femoral head from the weight-bearing portion, decreasing the load subjected to infarction in order to prevent collapse in the early stage. The key point of CVO is to create a curved varus osteotomy line though the tip of great trochanter and the middle of the lesser trochanter without exceeding the intertrochanteric crest. The osteotomy center is not necessarily located in the femoral head center [13].

Clinically, to estimate the collapse risk after CVO, the intact ratio is used and defined as the intact surface area divided by the weight-bearing area in the femoral head. A post-operative intact ratio of 33.0% was necessary if a satisfactory result was to be achieved [14], whereas it was prone to collapse if the necrotic lesion was located in the anterolateral weight-bearing portion or with insufficient femoral head coverage [15]. Moreover, the post-operative intact ratio of CVO <33.3% and lateral center-edge (LCE) angle <25° were identified as independent factors leading to radiographic failure and conversion to total hip arthroplasty from clinical reports [16]. However, from a biomechanical viewpoint, few studies have focused on the stress changes of necrotic bone and the influence of different LCE angles on the stress distribution of necrotic bone after CVO. In addition, the correlation between the intact ratio and stress in the necrotic bone has not been discussed.

A finite element analysis (FEA) is a popular computer simulation for evaluating the stress and strain state in the human body, including studies on ONFH [9, 17–20]. Thus, the purpose of the present study was to evaluate the influence of CVO on the stress in the necrotic femoral head using an FEA. We also evaluated the effect of the LCE angle on the stress distribution in the segment of necrotic bone for better decision-making in relation to CVO.

## Methods

This biomechanical study was approved by our ethical review committee (NO. 0-0672). A healthy volunteer (sex: male, age: 27 years, height: 164 cm, body weight: 66kg) without any musculoskeletal disease or history of hip joint operations was recruited. Quantitative computed tomography (QCT) was scanned in combination with a calibration phantom (B-MAS200, Kyoto-kagaku, Kyoto, Japan). The resolution of each CT image was 512 by 512 pixels with a slice thickness of 1.0 mm, and the pixel size was 0.782 mm/pixel under 120 KV and 102.50 mA conditions.

### CVO model with ONFH

**Fig. 1:**
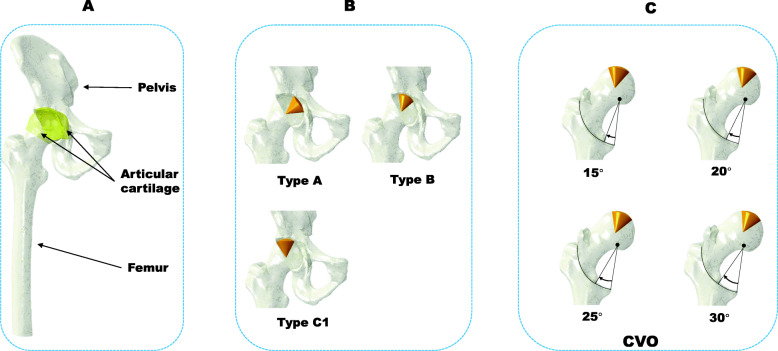
Construction of the CVO model with femoral head necrosis. **a** The hip joint model extracted from CT data. **b** The three locations with a cone angle of 60°: type A, type B, and type C1. **c** Intertrochanteric cured varus osteotomy with a rotation angle of 15°, 20°, 25°, and 30°

The intact hip joint model was constructed from the QCT data by segmenting the bony structure of the ilium and femur using a medical image processing software program (Mimics 22, Materialise, Belgium) (Fig. 1a). The cartilage geometry was created by filling the clearance gap between the acetabulum and the femoral head. Then, the cartilage model was divided into acetabular and femoral head cartilage with a spherical surface [21].

The necrotic bone model was created based on the Japanese Investigation Committee (JIC) classification [22]. The size of the necrotic lesion was set as a conoid shape (cone angle: 60°) to represent an early stage, low risk, and pre-collapse of the lesion [18]. The typical three locations of the necrotic bone (types A, B, C1) were simulated. (Fig. 1b). In brief, the weight-bearing portion of the acetabulum was trisected; the vertical line through lateral node of each aliquot reached the point over the femoral head that was defined as the boundary of the lateral locations of types A, B, C1 in the central coronal section of the femoral head of CT images. The locations matched with the changes of the weight-bearing portion, which were decided by different LCE angles in this study.

The CVO models were created using a CAD system (SolidWorks 2016, SolidWorks Corp., USA) (Fig. 1c). The osteotomy plane was set from the frontal view, and the femur model was cut along the plane by Boolean operations. The osteomized proximal part was rotated and fixed to the distal femur with four different angles of 15°, 20°, 25°, and 30° without implants.

### 3D Measurement of geometric parameters

**Fig. 2:**
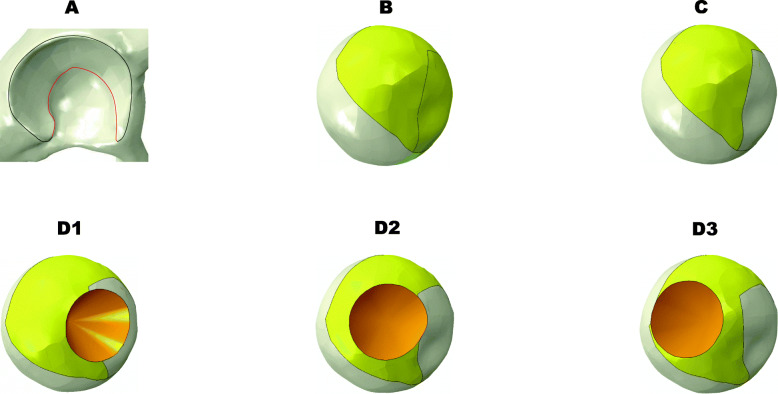
Three-dimensional measurement of the femoral head coverage, weight-bearing area, and intact area. **a** The acetabular rim line (in black) for determining the acetabular coverage and acetabular arch line (in red) for determining the weight-bearing area. **b** The acetabular coverage projected on the femoral head in yellow. **c** The weight-bearing area projected on the femoral head in yellow. **d1–d3** The intact area in yellow of types A, B, and C1 was determined by removing the necrotic area in orange (top view)

Femoral head coverage area was measured in each hip joint model [23]. The spline curve was fit to the rim of the acetabulum (Fig. 2a, black line, lateral to medial) and projected on the surface of the femoral head to create an area of femoral head coverage (Fig. 2b). The weight-bearing area was defined as the lunate surface in this study. The border curve between the lunate surface and the acetabular fossa (Fig. 2a, red line) was projected on the surface of the femoral head to create the weight-bearing area (Fig. 2c). The intact area was defined as the area with the necrotic region removed from the weight-bearing area (Fig. 2d). The intact ratio was calculated as the intact area/the weight-bearing area. The results of the measurement are summarized in (Table 1).

**Table 1:** 3D-Measurement of geometrical parameters

Necrosis	CVO angle	FHC (mm^2^)	WBA (mm^2^)	Intact area (mm^2^)	Intact ratio
Type A	0	2776.06	1971.59	1795.18	0.91
	15	2808.67	2001.17	1927.44	0.96
	20	2816.22	1998.32	1952.64	0.98
	25	2856.75	2015.33	1993.78	0.99
	30	2874.13	2018.79	1997.84	0.99
Type B	0	2776.06	1971.59	1502.76	0.76
	15	2808.67	2001.17	1635.54	0.82
	20	2816.22	1998.32	1677.76	0.84
	25	2856.75	2015.33	1749.82	0.87
	30	2874.13	2018.79	1834.20	0.91
Type C1	0	2776.06	1971.59	1457.45	0.74
	15	2808.67	2001.17	1497.21	0.75
	20	2816.22	1998.32	1507.74	0.75
	25	2856.75	2015.33	1560.04	0.77
	30	2874.13	2018.79	1597.72	0.79

*FHC* femoral head coverage, *WBA* weight-bearing area

### Material properties assignment and boundary configurations

**Fig. 3:**
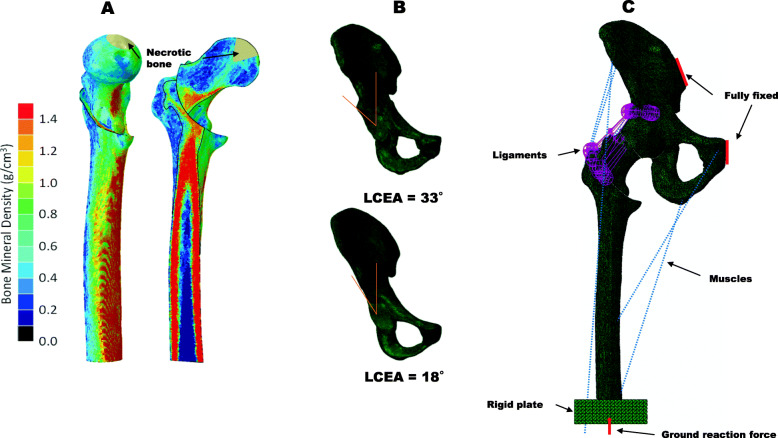
The finite element model of CVO. **a** The femur was mapped with isotropic heterogeneous material properties based on grayscale values from the QCT data. **b** Lateral center-edge angle = 33° to represent the normal weight-bearing portion and lateral center-edge angle = 18° to represent the insufficient weight-bearing portion. **c** Ground reaction force was imposed at the distal end of the femur (red arrow); several muscles (blue dotted lines) and the ligaments (purple lines) around the hip joint were attached; the interfaces of the CVO were bounded without fixation

The hip joint models were meshed 928,127 four-node tetrahedral elements with an element size of approximately 1 mm for bone and cartilage, which are assumed to be isotropic and elastic materials. For the femur model, heterogeneous Young’s modulus was assigned to each element based on QCT data (Fig. 3a) [24]. Briefly, the parameters used for converting Hounsfield Units (HU) to radiographic CT density (*ρ*_*QCT*_(g/cm^3^) (Eq. (1)) were calculated from the B-MAS200 phantom [25] and from *ρ*_*QCT*_ to Ash density (*ρ*_*ash*_(g/cm^3^) (Eq. (2)) [26], then the apparent density that was calculated from the Ash density with a ratio of 0.6 [27] was converted to the elastic modulus (Eq. (3)) [28].

1 $$ {\rho}_{QCT}\left(\mathrm{g}/{\mathrm{cm}}^3\right)=0.9863\mathrm{HU}\hbox{-} 2.0804 $$

2 $$ {\rho}_{ash}\left(\mathrm{g}/{\mathrm{cm}}^3\right)=0.877\times {\rho}_{QCT}+0.0789 $$

3 $$ E=6850{\rho}_{app}^{1.49} $$

The necrotic lesion, cartilage and ilium were assigned with homogeneous material properties based on the literature (Table 2) [18, 29, 30].

**Table 2:** Material properties for FE model

Component	Elastic modulus (MPa)	Poisson’s ratio
Femur bone	7.173–15769.50	0.3
Pelvis bone	17000	0.3
Necrotic bone	124.6	0.152
Articular cartilage	10.5	0.45

To simulate the standing loading, a ground reaction force of 700 N was applied to a rigid plate fixed to the distal end of the femur. Seven muscles around the hip joint were modeled using connector elements based on a previous study (Table 3) [29]. The forces can minimize the bending moment in every cross-section of the femur [31]. The hip capsular ligaments were modeled as a 1D springs element. The stiffness of the superior iliofemoral, inferior iliofemoral, ischiofemoral, and pubofemoral ligaments were set at 96, 102, 40, and 36 N/mm, respectively. The width of ligament was simulated by several springs; the numbers of springs was adopted from a previous study [32]. The stiffness of each spring was calculated as the stiffness of ligament divided by the number of springs. The position of origin and insertion of springs was determined at the surface nodes of bone and mimicked the anatomical position of the ligament as much as possible [33]. The pubic symphysis and sacroiliac joint were fully fixed to prevent translation and rotation. The osteotomy interface was set as a bond, and the contact area of cartilage was modeled as frictionless.

**Table 3:** Loading for finite element model

Component	Forces (N)	Component	Forces (N)
Adductor longus	560	Gluteal minimus	300
Adductor magnus	600	Piriformis	500
Gluteal maximus	550	Tensor fascia latae	300
Gluteal medius	700	Ground reaction force	700

We also modified the acetabulum shape to investigate the influence of the weight-bearing portion on the stress state in the necrotic lesion after CVO (Fig. 3b). The model with an LCE angle of 33° represented the normal weight-bearing portion, and the model with an LCE angle of 18° represented the insufficient portion [34, 35]. Finally, a total of 30 different finite element models were created simulating three locations of necrosis combined with four CVO angles in two different LCE angle conditions (Fig. 3c). The FE analysis was performed using a general-purpose FEA software program (ABAQUS 2019, Dassault Systems, Providence, RI).

### Sensitivity studies and mesh convergence tests

Changes in the assumed material properties were performed to investigate how such properties affect the stress and strain predictions for sensitivity studies in physiological conditions using the intact hip joint model. We analyzed the variations in the FE model prediction, when the elastic modulus of the pelvis bone and articular cartilage were changed to ±10 % of the initial values [30]. The results of the sensitivity studies are presented in Table 4. In our selected models, the variation of the sensitivity studies was confirmed to be <5% by evaluating the maximum von Mises stress of the femur. This information demonstrated that the alteration in material parameters did not result in significant changes. According to the modulus and Poisson’s ratios, the FE-predicted stress was relatively insensitive to change [36]. This indicates that the material coefficient used in the study was stable and effective.

**Table 4:** Sensitivity studies of FE models

Models	Pelvis bone	Cartilage	Max. von Mises stress (MPa)
1	*E* = 17 GPa *v* = 0.3	*E* = 10.5 MPa *v* = 0.45	298.50739
2	*E* = 17 GPa *v* = 0.3	*E* = 11.55 MPa *v* = 0.45	298.50583
3	*E* = 17 GPa *v* = 0.3	*E* = 9.45 MPa *v* = 0.45	298.50977
4	*E* = 18.7 GPa *v* = 0.3	*E* = 10.5 MPa *v* = 0.45	298.50909
5	*E* = 15.3 GPa *v* = 0.3	*E* = 10.5 MPa *v* = 0.45	298.50528

*E* elastic modulus, *v* Poisson’s ratio

Mesh convergence tests were performed to confirm the discretization for the analysis. A mesh was generally considered to be sufficiently refined when an increase in mesh resolution yielded less than a 5% change in the result [37]. The number of tetrahedral elements in the intact femur model was increased to analyze the stress variation by evaluating the maximum von Mises stress of the femur (Table 5). Based on the convergence test, the elements of 928,127 with an average element size of 1 mm (percentage variation within 5%) was chosen as the final FE model to minimize the discretization error for the verification of the calculation [38].

**Table 5:** Convergence tests of FE models

Models	Elements no.	Max. von Mises stress (MPa)
1	516060	298.50555
2	553764	298.50919
3	585758	298.50858
4	759401	298.50708
5	928127	298.50739
6	993256	298.50757

## Results

### The effectiveness of CVO on stress reduction in necrotic bone

**Fig. 4:**
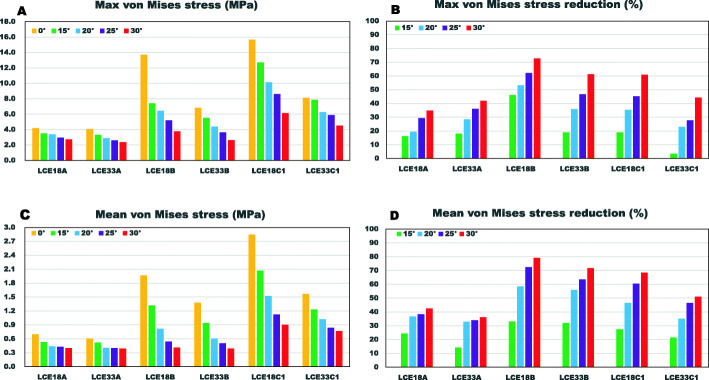
Von Mises stress in different models. **a** Maximum von Mises stress of the models. **b** The maximum von Mises stress reduction after CVO. **c** The mean von Mises stress of the models. **d** The mean von Mises stress reduction after CVO

**Fig. 5:**
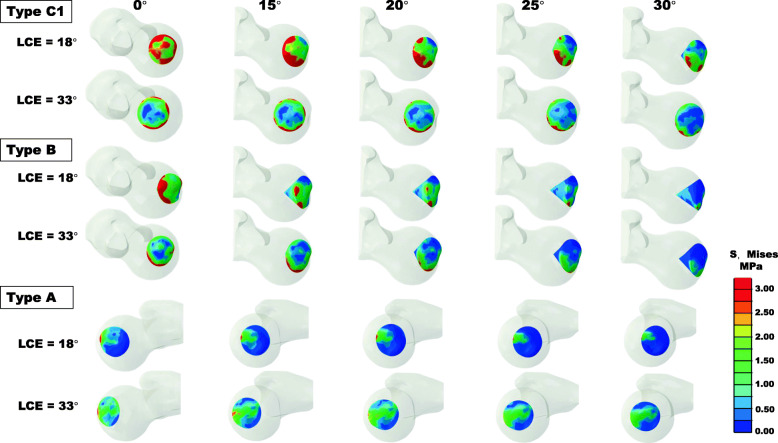
Top view of the von Mises stress distribution of types A, B, and C1 in two different weight-bearing portions with several CVO angles

**Fig. 6:**
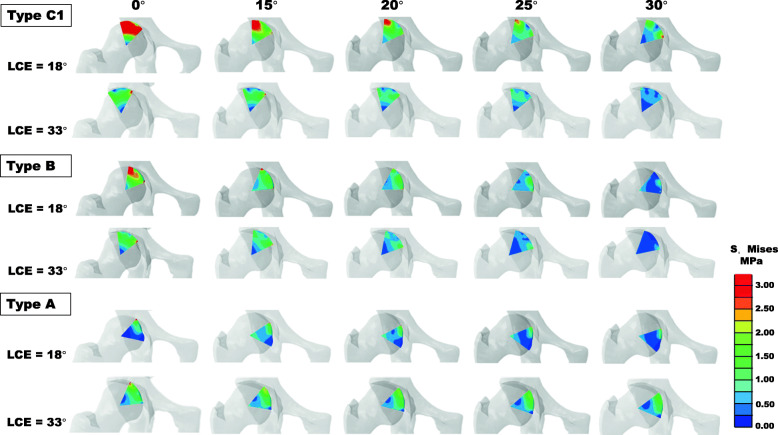
Coronal central section view of the von Mises stress distribution of types A, B, C1 in two different weight-bearing portions with several CVO angles

The maximum and mean von Mises stress in the necrotic bone were reduced with an increase in the CVO angle (Fig. 4). In particular, in the insufficient weight-bearing models (LCE angle of 18°), the maximum stress was reduced from 15.67 to 6.13 MPa for type C1 and from 13.73 to 3.76 MPa for type B (Fig. 4a). Correspondingly, the stress concentration at the interface of healthy and necrotic bone was weakened, accompanied by an increase in the CVO angle (Figs. 5 and 6). For type A, with both LCE angles, slight stress reduction was observed after CVO in comparison to types B and C1 (Fig. 4b, d).

### Influence of the weight-bearing portion

The LCE angles affected the maximum and mean stress in the necrotic bone, with the exception of type A. The stress with an LCE angle of 18° were higher in comparison to the stress with an LCE angle of 33°, for types B (maximum stress: 13.73 vs 6.83 MPa) and C1 (maximum stress: 15.67 vs 8.14 MPa) in CVO 0° models (Fig. 4a). With an LCE angle of 18°, extensive stress distribution was observed in the necrotic region in comparison to an LCE angle of 33° (Figs. 5 and 6).

### Relationship between geometric measurements and stress of necrosis

**Fig. 7:**
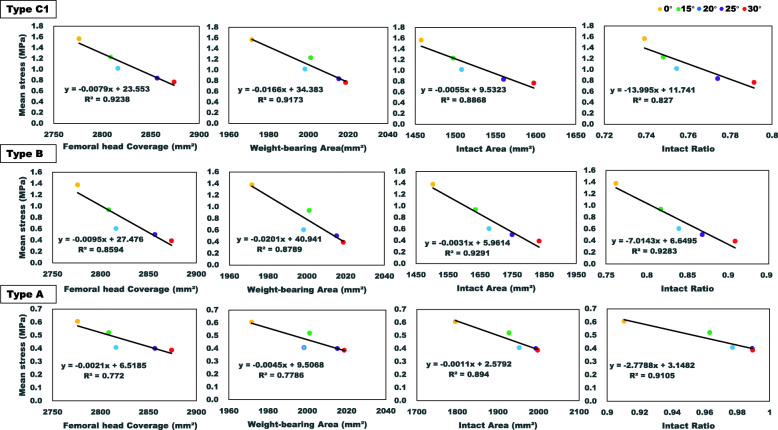
The correlation of femoral head coverage, weight-bearing area, intact area, and intact ratio with the mean von Mises stress of necrosis of types A, B, and C1

The correlation of these geometric measurements with the mean von Mises stress of the necrotic region was compared (Fig. 7). From the results of this linear regression analysis, the geometric measurements and stress had a negative relationship with good fitness, as determined by the *R*^2^ value. The stress of necrosis decreased by increasing the intact ratio, intact area, weight-bearing area, and femoral head coverage. However, the intact ratio had more influence on the stress of the necrotic bone in comparison to other geometric measurements with a large slope value.

## Discussion

Proximal femoral osteotomy for the treatment of ONFH is performed with the aim of eliminating the mechanical factors suffered by necrotic bone. This is why the mechanical simulation of such procedures is necessary for assisting preoperative decision-making [9]. In this study, the biomechanical effectiveness of CVO was addressed by evaluating the stress of necrotic bone after CVO (Fig. 4). All types of necrotic lesions would mechanically benefit from CVO with different levels of stress reduction. As expected, in the case of types B and C1, the higher degree of CVO performed, the greater the degree of stress reduction that was achieved, because the necrotic bone is removed from the weight-bearing portion to a medial site by varus osteotomy. For example, the maximum stress of type C1 from 8.14 MPa in 0° situation was reduced to 4.53 MPa after a CVO of 30° with a maximum stress reduction of 44.3% (Fig. 4a, b). These results suggested that CVO was an effective way to reduce the stress level in necrotic bone.

Our simulation demonstrated that the appropriate CVO angle depended on the type of necrotic lesion. For type A, there was little change in the stress after CVO, and the stress level was relatively lower in 0° models. This result can support the clinical repots [7, 14, 16, 39–44]. Therefore, it was not effective to perform the CVO with type A. For type B, satisfactory results could be achieved from 20° (0.61 MPa), and for type C1, an angle of at least 30° (0.77 MPa) was considered safe, if the stress level of type A (0.61 MPa) was regarded as a standard, based on the results predicted in this study.

Unfavorable biomechanical situation of the hip such as acetabular dysplasia, which can increase contact stress as well as has been identified as a risk factor for the collapse of necrotic bone, leads to highly nonuniform contact stress distribution in comparison to the normal hip joint [16]. In this study, the stress in the insufficient weight-bearing portion models was higher in comparison to normal weight-bearing models. In the case of type C1, the mean stress value was 2.85 MPa of LCE angle 18° vs. 1.57 MPa of LCE angle 33° in the no osteotomy model, and it was higher (0.90 MPa vs 0.77 MPa) in the LCE angle 18° model even after CVO 30°. Type A had received the least influence from the weight-bearing portion (Fig. 4c). From these results, the influence of the weight-bearing portion on stress distribution of lesion was location-dependent, the more lateral site located, the more stress suffered. Thus, further procedures, such as periacetabular osteotomy, should be considered, when CVO is performed for a hip with an unfavorable biomechanical situation.

Stress concentrations were observed at not only the lateral interface region but also anterior region for types B and C1 (Figs. 5 and 6), which indicated that the anterior side was also at risk of collapse. The finding was supported by a clinical report on CVO [39]. This report suggested that the anterior necrotic angle was associated with the progressive collapse of the anterior lesion.

The intact ratio is used as the most important indicator for evaluating the risk of collapse after CVO in the clinical setting [14]. It is calculated from the transposed intact articular surface of the femoral head and the weight-bearing area of the acetabulum on anteroposterior radiographs [39]. However, the relationship between the intact ratio measured in 3D and the stress of necrotic bone after CVO was firstly discussed in this study. We found that a strong negative correction between the intact ratio and the stress level of necrotic bone after CVO had more influence on stress distribution with a larger slope value in the linear regression model, in comparison to the other parameters of femoral head coverage, weight-bearing area, and intact area (Fig. 7). Thus, it was confirmed that the intact ratio could be a bridge between the clinical results and the stress reduction of necrosis after CVO.

The present study was associated with some limitations. (1) Only one healthy subject was evaluated; thus, the deviation derived from patient-specific differences could not be considered [45, 46]. The study should have investigated additional types of necrosis from patient data. (2) Finite element models of physiological loading conditions should generally be validated from electromyography data [47–49]. For the present study, validation of the predicted results depended on similarity to the physical phenomenon of the real structure of femoral head with an intact model subjected to loading. (3) The interfaces of the CVO were assumed to be fully bonded, which more likely simulates the healed stage after CVO without taking the influence of the fixation device into account when simulating the initial postoperative stage. (4) The mechanical property of human articular cartilage displayed a viscoelastic and nonlinear mechanical response to loading [46, 50]; however, an isotropic homogeneous material property was assigned in the study [29, 35]. (5) This simulation only addressed mechanical factors that would affect the stress distribution of necrotic bone; however, biological reaction that may be different after each CVO also needs to be considered.

## Conclusions

Understanding the complicated interdependence of the size and location of necrotic lesions and the configuration of the hip and pelvis is important in making decisions regarding optimal treatment. This finite element study suggested all types of lesions would mechanically benefit from CVO. However, for type B, an angle of >20° was recommended, and for type C1, an angle of at least 30° was safe, decreasing to the stress level of type A. An insufficient weight-bearing portion at acetabulum such as dysplasia can lead to the stress concentration at the necrotic bone rather than the normal hip joint. The intact ratio could be a bridge between the clinical outcomes and stress level in CVO.

## Data Availability

The data and materials are available from the corresponding author.

## Abbreviations

CVO: Curved varus osteotomy
ONFH: Osteonecrosis of the femoral head
LCE: Lateral center-edge angle
FEA: Finite element analysis
QCT: Quantitative computed tomography
JIC: Japanese Investigation Committee
HU: Hounsfield Unit
Eq: Equation
CAD: Computer-assisted design
vs: Versus
3D: Three-dimensional
